# Rare Variants Cause Charcot‐Marie‐Tooth Disease in Malian Families

**DOI:** 10.1002/brb3.70496

**Published:** 2025-05-05

**Authors:** Abdoulaye Yalcouyé, Lassana Cissé, Salimata Diarra, Seybou H. Diallo, Salia Bamba, Patra Yeetong, Boubacar Maiga, Kékouta Dembélé, Dramane Coulibaly, Salimata Diallo, Abdoulaye Taméga, Alassane Baneye Maiga, Hamidou O. Ba, Vorasuk Shotelersuk, Kenneth H. Fischbeck, Cheick O. Guinto, Guida Landouré

**Affiliations:** ^1^ Faculté de Médecine et d'Odontostomatologie USTTB Bamako Mali; ^2^ McKusick Nathans Institute and Department of Genetic Medicine Johns Hopkins University Baltimore Maryland USA; ^3^ Service de Médecine Hôpital Nianankoro Fomba de Ségou Ségou Mali; ^4^ Neurogenetics Branch NINDS, NIH Rockville Maryland USA; ^5^ Service de Neurologie Centre Hospitalier Universitaire “Gabriel Touré” Bamako Mali; ^6^ Division of Human Genetics, Department of Botany, Faculty of Science Chulalongkorn University Bangkok Thailand; ^7^ Service de Médecine Centre Hospitalier Universitaire “Le Luxembourg” Bamako Mali; ^8^ Service de Cardiologie Centre Hospitalier Universitaire “Gabriel Touré” Bamako Mali; ^9^ Center of Excellence for Medical Genomics, Department of Pediatrics, Faculty of Medicine Chulalongkorn University Bangkok Thailand; ^10^ Excellence Center for Genomics and Precision Medicine King Chulalongkorn Memorial Hospital, the Thai Red Cross Society Bangkok Thailand; ^11^ Service de Neurologie Centre Hospitalier Universitaire du Point G Bamako Mali

**Keywords:** Africa, *BAG3*, *BSCL2*, Charcot‐Marie‐Tooth disease, Mali, *PEX10*, *SH3TC2*

## Abstract

**Introduction/Aims:**

Charcot‐Marie‐Tooth disease (CMT), the most common inherited peripheral neuropathy, is clinically and genetically heterogeneous with over 100 genes identified to date. Recently, next‐generation sequencing (NGS) has enabled molecular diagnosis in previously unidentified CMT cases. However, less progress has been achieved in sub‐Saharan African (SSA) populations. We report rare CMT variants found in four unrelated Malian families.

**Methods:**

Patients went through a thorough neurological examination and Nerve Conduction Studies (NCS) were performed. DNA was extracted for genetic analysis (CMT gene panel testing and whole‐exome/genome sequencing). Putative variants were confirmed with Sanger sequencing and segregation was checked in all available family members. Deleteriousness was checked using several in silico prediction tools and protein modeling.

**Results:**

Nine patients (three males and six females) from four families were enrolled. The mean age at onset and diagnosis were 15 and 22.7 years, respectively (ranges: 3 to 55 years, and 12 to 58 years). Walking difficulty was the first symptom commonly reported. Neurological examination found distal muscle weakness and wasting with sensory loss, reduced tendon reflexes, and skeletal deformities. In addition, some patients presented with ataxic gait associated with incoordination that are not in the forefront of CMT features. NCS was consistent with the axonal pattern in three families. Genetic analysis revealed rare pathogenic variants in *BSCL2*, *SH3TC2*, and *PEX10*, and of unknown significance in *BAG3*.

**Discussion:**

This study reports rare variants in these CMT genes for the first time in SSA populations, expanding the global epidemiological, clinical, and genetic spectrum of these diseases.

## Graphical abstract

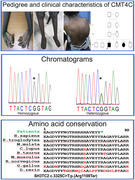

## 1 Introduction

Charcot‐Marie‐Tooth disease (CMT), also known as hereditary motor and sensory neuropathy, is the most common inherited neuromuscular disorder with an estimated prevalence of 1/2500 worldwide (Skre 1974). CMT is a clinically and genetically heterogeneous umbrella of neurodegenerative diseases that lead to the loss of motor and sensory functions of the peripheral nerves (Stavrou et al. 2021). Typically, CMT phenotype includes distal muscle weakness and atrophy, sensory loss, abnormal tendon reflexes, and skeletal deformities. In rare cases, other manifestations, including vocal cords and cranial nerve paralysis, hearing or vision loss, and pyramidal and cerebellar symptoms may be seen, though not often in the forefront of CMT features (Bird 2025; Landouré et al. 2010). Electro‐physiologically, CMT is divided into two main groups, demyelinating type (CMT1 and CMT4) and axonal type (CMT2), based on the median nerve conduction velocities (Reilly et al. 2011). To date, over 100 genes are associated with CMT phenotypes, of which at least 90% of molecularly diagnosed cases are caused by variants in four genes, including *PMP22*, *GJB1*, *MFN2*, and *MPZ* genes (Pipis et al. 2019). All Mendelian patterns have been described, with autosomal dominant being the most prevalent, globally (Reilly et al. 2011). With the recent advancement of next‐generation sequencing (NGS), several novel CMT gene variants were identified (Pipis et al. 2019). Importantly, most CMT cases have been described in Eurasians while data from African populations are notably scant (Yalcouyé et al. 2022). However, family‐based studies have reported known and some novel genetic findings, including *GJB1*, *GARS*, and *CADM3* in Mali (Yalcouyé et al. 2009, 2005, 2023). In this study, we investigated four unrelated Malian families with CMT phenotypes in which we identified rare variants in *BAG3, BSCL2, SH3TC2*, and *PEX10*.

## 2 Methods

This study was conducted in full compliance with the declaration of Helsinki. Institutional ethical approval (N°2018/182/CE/FMOS) was obtained from the Faculty of Medicine and Dentistry, Bamako, Mali.

Comprehensive clinical assessment was performed by neurologists, cardiologists, ophthalmologists, and ear, nose, and throat (ENT) specialists. Blood chemistries were performed to exclude common causes of acquired polyneuropathies. In addition, creatine kinase and lactate dehydrogenase dosage, electrocardiogram, and echocardiography were done, where needed. Nerve conduction studies (NCS) were performed at least in the proband of each family to refine the phenotype characterization. DNA was extracted from the peripheral blood of all available participants for genetic analysis. Next‐generation sequencing CMT gene panel testing (a list of the genes is provided in Table S1, which includes 50 genes and mtDNA) was performed at Medical Neurogenetics, Atlanta, GA (MNG Laboratories) in Families 1 and 2. Whole‐exome sequencing (WES) was performed in Families 3 and 4, and Whole‐genome sequencing (WGS) using single molecule real‐time (SMRT) sequencing with Pacbio HIFI technology was subsequently performed in Family 4. Variants were confirmed with Sanger sequencing, and segregation analysis was performed on all available family members. Various in silico prediction tools were used to assess the deleteriousness of the putative variants and were finally classified following the guidelines of the American College of Medical Genetics and Genomics (ACMG) (Yalcouyé et al. 2022; Richards et al. 2015). Moreover, analyses of the secondary and three‐dimensional (2D and 3D) structures were performed using the AlphaFold 3 server to investigate structural changes in the mutant protein (Abramson et al. 2024). More details are provided in the supplementary methods.

## 3 Results

### 3.1 Family 1

**FIGURE 1:**
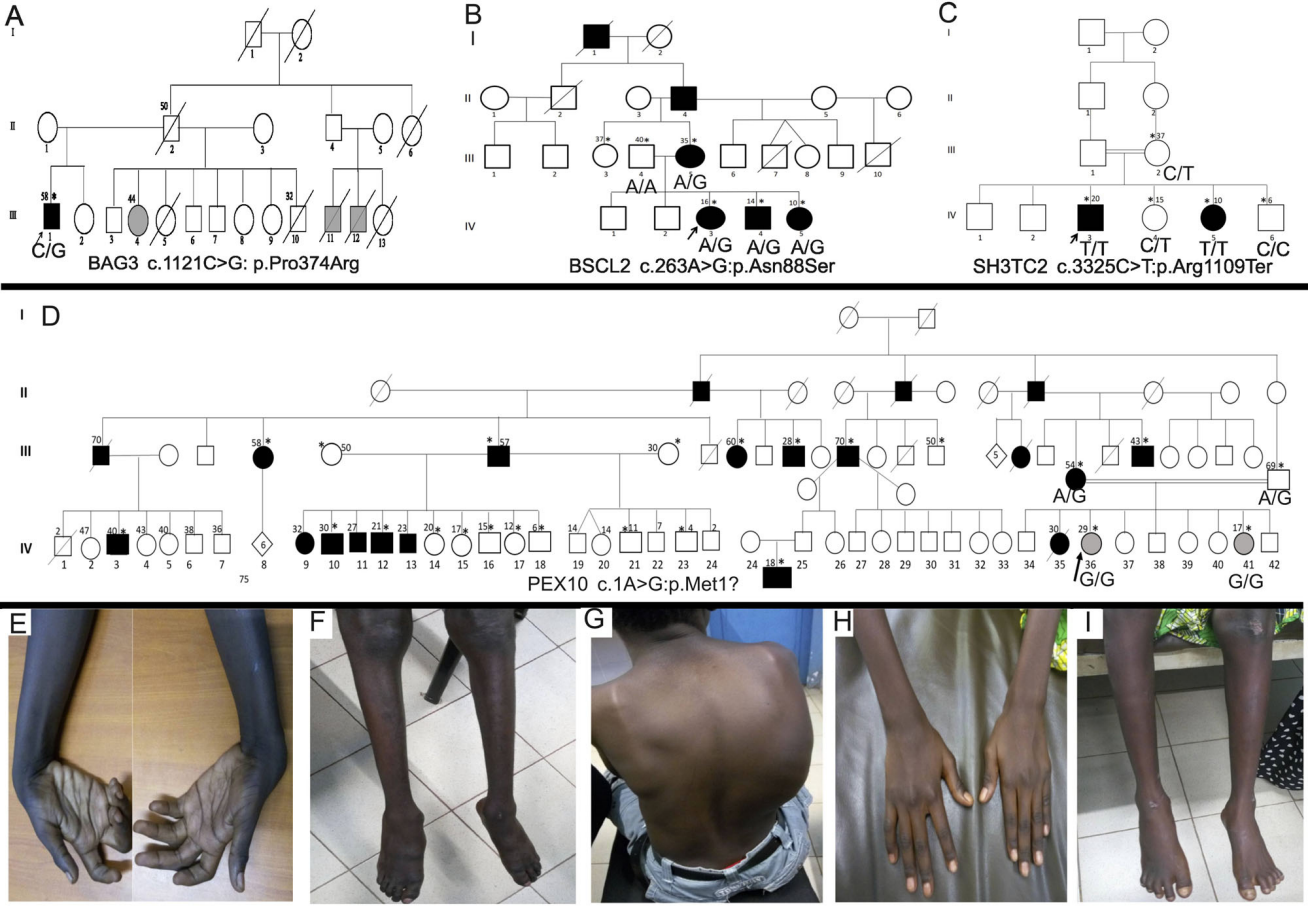
Pedigrees and phenotypic characteristics of CMT families. (A) Pedigree of Family 1 with *BAG3*‐neuropathy. Affected individuals are represented with black/grey squares. (B) Pedigree of family 2 with *BSCL2*‐neuropathy showing an autosomal dominant inheritance pattern. Affected individuals are represented with a black square and circle. (C) Pedigree of Family 3 with *SH3TC2*‐neuropathy showing an autosomal recessive inheritance with consanguinity (double horizontal line). Capital letters at the bottom are genotype information. (D) Pedigree of Family 4 with *PEX10*‐neuropathy showing an autosomal recessive inheritance pattern for the neuropathy phenotype with consanguinity (horizontal double line). Numbers on top stand for ages at examination, an arrow for proband, and an asterisk for those seen in the clinic. Grey‐filled circles are individuals sharing neuropathy and BAFME8 phenotypes, and black‐filled circles and squares are those having only the BAFME8 phenotype. For all pedigrees, numbers on top stand for ages at examination, an arrow for proband, an asterisk for those seen in the clinic, and capital letters in the bottom are genotype information. (E–G) Images of patient IV.3 of Family 3 showing distal muscle atrophy in four limbs and dorsal scoliosis. (H, I) Images of patient IV.5 of Family 3 showing less severe phenotype.

**FIGURE 2:**
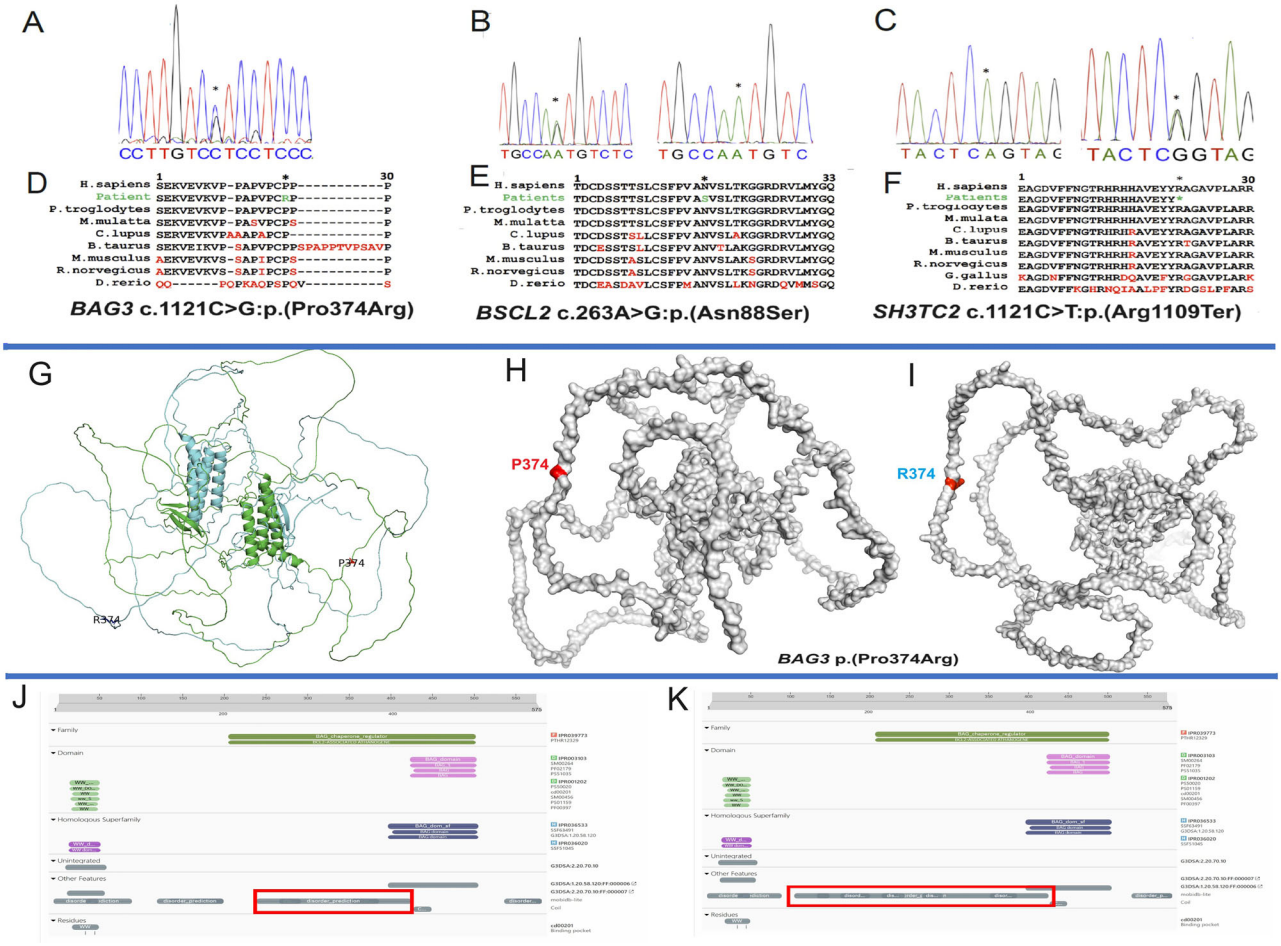
Genetic and three‐dimensional structures data. (A) Electropherogram showing the heterozygous nucleotide “C” to “G” change in *BAG3*, (B) Electropherogram showing the heterozygous nucleotide “A” to “G” change and a normal sequence in *BSCL2*, (C) Electropherogram showing the homozygous nucleotide “C” to “T” change and a carrier in *SH3TC2*, (D) Portion of BAG3 protein showing the conservation of Pro374 across a wide range of species, (E) Portion of BSCL2 protein showing the conservation of Asn88 across a wide range of species, (F) Portion of SH3TC2 protein showing the conservation of Arg1109 across a wide range of species, (G–I) Superimposed structure of wildtype (green) and mutant BAG3 (light blue) protein showing the three‐dimensional conformational changes in the mutant compared to the wildtype. (J, K) InterPro protein domain search showing the extension of the disordered region of the protein in the mutant compared to the wild type.

A 58‐year‐old male (III.1) from a nonconsanguineous family of Bambara ethnicity was referred to our clinic for walking difficulty (Figure 1A). His past medical history was consistent with high blood pressure over 10 years, and his two paternal cousins and half‐sister reportedly present similar symptoms. The disease started at the age of 55 years with walking difficulty that worsened gradually, associated with tingling, muscle cramps, and weakness in distal limbs. Neurological examination found distal muscle weakness and atrophy, sensory loss in four limbs, predominating in lower limbs. Tendon reflexes were absent in lower limbs and reduced in upper ones, and a bilateral valgus big toe deformity was noted. In addition, he presented with a steppage gait. The total creatine kinase (1218 UI/L; Normal: 29–200 UI/L) and lactate dehydrogenase (549 UI/L; Normal: 228–456 UI/L) were elevated. Echocardiography was normal, but the electrocardiogram suggested left ventricular hypertrophy. Nerve conduction studies showed an axonal type length‐dependent neuropathy with reduced amplitudes in nerve conduction velocity (NCV). Next‐generation sequencing CMT gene panel testing revealed a heterozygous missense variant, c.1121C> G: p.(Pro374Arg) in the *BAG3* gene (#NM_004281.4), confirmed with Sanger sequencing (Figure 2A). The Pro374 residue is conserved across various species (Figure 2D). More clinical and laboratory details are provided in Table 1. Several in silico tools predict this variant as deleterious (CADD = 19.9) and is classified as a variant with unknown significance according to ACMG criteria (Table S2). Interestingly, 2D structure prediction showed significant alterations involving the helical structure located at ^125^AA^126^ and the beta sheets at ^46^TTWN^50^ and ^96^IPV^98^, leading to conformational sift, which were apparent in 3D modeling (Figure 2G–I; Figure S1A,B) with extension of the disordered region of the mutant protein (Figure 2J & K) (Blum et al. 2025).

**TABLE 1:** Clinical, electrophysiological, and genetic characteristics of patients with Charcot‐Marie‐Tooth disease.

		Clinical characteristics																		Nerve conduction studies				Genetic data	
**Family**	**Patients**	Age	Sex	Age of onset	First symptoms	Motor signs					Sensory signs		Other symptoms	Disability status	Motor median			Ulnar		Sural		Sensory median		Genes	Variants
						Muscle weakness	Tendon reflexes	Muscle atrophy		Skeletal deformities	Touch and pinprick	Vibration and joint position sense			CMAP Amp (mV)	CV m/s	CMAP Amp (mV)		CV m/s	SNAP Amp	CV m/s	CV m/s	SNAP Amp (uV)		
						UL/LL	UL/LL	UL	LL		UL/LL	UL/LL													
1	**III.1**	58	M	55	Hands weakness and tingling	++/+++	↓/‐	Thenar, hypothenar, interosseous	Peroneal, EDB	Bilateral valgus big toes	↓/↓	↓/↓	—	Walk with a cane	8.5	44	7.6		55	NR	NR	NR	NR	*BAG3*	c.1121C> G: p.Pro374Arg
2	**III.5**	35	F	25	Walking difficulty	++/+++	N/↓	Thenar, hypothenar	Peroneal	Hammer toes and claw hands	↓/↓	N/↓	—	Walk unaided	ND	ND	ND		ND	ND	ND	ND	ND	*BSCL2*	c.263A> G:p.Asn88Ser
	**IV.3**	16	F	10	Walking difficulty, paresthesia, and pain	+++/+++	‐/‐	Thenar, hypothenar, interosseous	Anterior leg	*pes cavus*, hammer toes and claw hands	↓/↓	↓/↓	—	Walk unaided	NR	NR	3.6		71	NR	NR	63	29		
	**IV.4**	14	M	7	Walking difficulty	+/++	N/N	—	—	Ankle deformation	N/↓	N/N	—	Walk unaided	ND	ND	ND		ND	ND	ND	ND	ND		
	**IV.5**	12	F	10	Walking difficulty	‐/+	↑/↑	—	—	—	‐/‐	‐/‐	—	Walk unaided	ND	ND	ND		ND	ND	ND	ND	ND		
3	**IV.3**	20	M	3	Walking difficulty	++/+++	↓/↓	Thenar, hypothenar, interosseous	Peroneal	Scoliosis, claw hands, pes planus	↓/↓	↓/↓	Balance disorder, dysmetria, dysarthria, ataxic and steppage gaits	Walk with assistance	2.8	4	NR		NR	NR	NR	NR	NR	*SH3TC2*	c.3325C> T:p.Arg1109Ter
	**IV.5**	10	F	3	Walking difficulty	++/++	↓/↓	Thenar, hypothenar, interosseous	Peroneal	—	↓/↓	↓/↓	Balance disorder, dysmetria, dysarthria, ataxic and steppage gaits	Walk unaided	2.2	6	1.2		7	NR	NR	NR	NR		
4	**IV.36**	32	F	17	Balance disturbance and leg pain	+/+	‐/‐	Interosseous	Peroneal	Hammer toes and pes cavus	N/N	N/N	Incoordination, slow eye movement, upper limb tremors, nystagmus, dysarthria	Walk unaided	15.7	57	11.9		57	4	55	59	7	*PEX10*	c.1A> G:p.Met1?
	**IV.41**	17	F	5	Walking difficulty	+/++	‐/‐	Interosseous	Peroneal	Hammer toes and pes cavus	↓/↓	↓/N	Upper limb tremors, dysmetria, nystagmus, ataxic gait	Walk unaided	21	53	11.3		54	17	54	58	34		

*Note*: normal median CMAP > 4.5 mV (recorded at abductor pollicis brevis muscle), Normal peroneal CMAP > 2.5 mV (recorded at extensor digitorum brevis muscle), normal tibial CMAP > 6 mV (recorded at abductor hallucis muscle), normal sural SNAP > 10 µV, normal F wave < 55 ms lower limbs and < 32 ms upper limbs, **#** both patients in this family also carry a pentanucleotide insertion in RAI1 gene causing Benign

Adult Familial Myoclonic epilepsy type 8 (BAFME8), ‐ absent, + slight, ++: moderate, +++: severe, ↑ brisk, ↓reduced/decreased.

Abbreviations: Amp, amplitude; CMAP, compound motor action potential; CV, conduction velocity; EDB, extensor digitorum brevis; LL, lower limbs; N, normal; ND, not done; NR, no response; SNAP, sensory nerve action potential; UL, upper limbs.

### 3.2 Family 2

Four affected individuals (one male and three females) and their unaffected relatives from a consanguineous family, of Bambara ethnicity were enrolled. However, the disease distribution was consistent with an autosomal dominant inheritance pattern (Figure 1B). The average age at onset was 13 years (range: 7–25 years) and at diagnosis was 19.3 years (range: 12–35 years), respectively. Symptoms started with a progressive waking difficulty reported in all affected individuals. Neurological examination found a distal muscle weakness ranging from slight to severe, predominating in the lower limbs, and distal muscle atrophy seen in older patients (III.5; IV.3) with sensory loss. Skeletal deformities including claw hands, hammer big toe, and *pes cavus* in three patients (III.5, IV.3, IV.4) were noted. Tendon reflexes were normal or decreased in these three patients while brisk in patient IV.5. Nerve conduction studies performed in the proband showed an axonal‐type length‐dependent neuropathy.

CMT gene panel testing identified a known heterozygous missense variant c.263A> G:p.(Asn88Ser) in the *BSCL2* gene (#NM_001122955.4), segregating with the phenotype within the family (Figure 2B). The Asn88 residue is located in a highly conserved domain of the protein (Figure 2E) and the p.Asn88Ser variant is predicted to be damaging by various in silico prediction tools, including CADD (score = 26.3; Table S2).

### 3.3 Family 3

Two affected individuals of Fulani ethnicity (one male and one female) from a consanguineous marriage and their healthy relatives were enrolled. The inheritance pattern was consistent with autosomal recessive (Figure 1C). Patients were aged 10 and 20 respectively at the time of diagnosis, and the disease began in their first decade of life with walking difficulty and frequent falls. Later, parents noticed skeletal deformities and dysarthria. Neurological examination found distal muscle weakness and atrophy predominating in lower limbs (Figure 1E–I), and sensory loss in four limbs. Tendon reflexes were decreased in both patients and patient IV.3 presented with dorsal scoliosis (Figure 2G). Furthermore, both patients presented balance disorder, dysmetria, and nystagmus in the lateral gaze predominating on the left side in patient IV.3 and multidirectional in patient IV.5. Additionally, they presented ataxic and steppage gaits (Video S1 of IV.5). Nerve conduction studies showed a demyelinating type neuropathy in the four limbs.

WES identified a known pathogenic homozygous variant c.3325C> T:p.(Arg1109Ter) in the *SH3TC2* gene (#NM_024577.4), segregating with the phenotype within the family (Figure 2C). The amino acid Arg1109 is located in a highly conserved region of the protein across a wide range of species (Figure 2F).

### 3.4 Family 4

Two female patients of Bambara ethnicity (aged 29 and 17 years old) from a consanguineous marriage and their family members were enrolled (Figure 1D). Symptoms started at the ages of 17 and 7 years old, respectively, with difficulty with balance and running, leading to frequent falls. Later, upper limb tremors, muscle weakness with difficulty in opening bottles, and dysarthria were noticed. Neurological examination found slight to moderate muscle weakness and atrophy, tremors, impaired coordination, and dysarthria. Patient IV.41 had a sensory loss in the lower limbs, horizontal bidirectional nystagmus with slightly slow eye movements. Skeletal deformities included hammer toes and *pes cavus* with ataxic gait in both patients. Nerve conduction studies showed axonal‐type length‐dependent neuropathy, more marked in the lower limbs. Their parents presented with tremors, walking difficulty, impaired coordination, and dysarthria on examination. However, distal neuropathy features were not reported during their examination.

Long read WGS (lrWGS) revealed a missense homozygous start loss variant c.1A> G:p.(Met1?) in the *PEX10* gene (#NM_001374425.1), in patients IV.36 and IV.41 only. Notably, these two patients also carried a heterozygote pentanucleotide repeat insertion in the *RAI1* gene causing Benign Adult Familial Myoclonic Epilepsy type 8 (BAFME8), while their parents had only the variant in *RAI1* (Yeetong et al. 2024). The variant p.(Met1?) segregates with the neuropathy phenotype within this family. in silico predictions showed this variant as damaging (CADD = 21.1) and classified it as pathogenic according to the ACMG criteria.

Clinical and laboratory findings of patients seen from all three families are summarized in Table 1.

Table S2 summarizes information about the in silico predictions.

## 4 Discussion

Over the past few decades, tremendous advances have been made to tackle CMT research (Rebelo et al. 2021; Rossor et al. 2020; Sahenk and Ozes 2020; Züchner and Pareyson 2022). However, a significant knowledge gap remains between developed countries and developing countries, particularly in SSA, where data on CMT are highly limited (Yalcouyé et al. 2022). Several novel genes and variants have been identified through collaboration including patients from underrepresented populations (Yalcouyé et al. 2023). In this study, we investigated four unrelated families from Mali, sharing CMT phenotypes caused by variants in *BAG3*, *BSCL2*, *SH3TC2*, and *PEX10* genes. Importantly, previous studies on various hereditary neurological disorders in Mali have also identified several novel variants and genes, including in CMT patients (Yalcouyé et al. 2019; Landouré et al. 2024, 2011; Traoré et al. 2009).

*BAG3* is associated with various conditions, including CMT2 and myofibrillar myopathy (Lee et al. 2012; Jaffer et al. 2012). However, only a few studies have reported CMT as the predominant feature in *BAG3‐*related disorders, similar to the family we describe here (Fu et al. 2020). Although the variant p.(Pro374Arg) is reported in the Genome Aggregation Database (gnomAD v4.1.0) with an allele frequency of 0.000001859, it was not found in individuals with black ancestry. In addition, due to the mild nature of some CMT phenotypes and their late onset features, these could be missed CMT cases, especially because this individual was not enrolled in a study screening neurological cases. Therefore, subtle signs could be missed. Jaffer et al. (2012), reported heterozygous *BAG3* mutations with clinical and histological evidence of giant axon neuropathy and further highlighted that neuropathy might be a predominant feature in *BAG3*‐related disorders. The conformational changes revealed by the 3D structure in the mutant protein could potentially disrupt BAG3 protein function. However, nerve or muscle biopsy was not available in our setting, but the muscular involvement was witnessed by the high CK levels as in previous reports (Kim et al. 2018). Further functional studies with cell or animal models will likely provide more insight regarding *BAG3* involvement in peripheral neuropathy.

*BSCL2* encodes for Seipin, an endoplasmic reticulum (ER)‐resident protein that is induced in the late stages of preadipocyte differentiation and is predicted to function in lipid droplet formation and/or metabolism (Cui et al. 2011). Yagi et al. (2011) demonstrated that p.(Asn88Ser) seipin mutant transgenic mice develop features of seipinopathy/*BSCL2*‐related motor neuron disease via ER stress. One patient in the family we describe here presented with brisk tendon reflexes, unlike other affected individuals in that family, suggesting pyramidal tract impairment. Previous studies have shown that patients with mutations in *BSCL2* show phenotypic variability, and pyramidal signs have commonly been reported in those patients, similar to our report (Ishihara et al. 2020).

*SH3TC2* encodes a protein expressed in Schwann cells of peripheral nerves and is localized to the plasma membrane and the perinuclear endocytic recycling compartment, suggesting its possible function in myelination and/or in regions of axoglial interactions (Arnaud et al. 2009). *SH3TC2* causes CMT4C, a severe recessive form of demyelinating CMT, characteristically presenting with skeletal deformities, including scoliosis and claw hands, as well as other symptoms such as nystagmus (Skott et al. 2019). The p.(Arg1109Ter) variant has been previously reported in European Gypsy families with a founder effect (Gooding et al. 2005). However, to the best of our knowledge, other signs or symptoms of cerebellar impairment, such as dysarthria and dysmetria have not been previously reported in patients carrying the p.(Arg1109Ter) variant. Additionally, CMT4C has been reported in North Africa by (Azzedine et al. 2006) with a similar phenotype. However, this is the first case of CMT4C in the SSA population.

*PEX10* encodes peroxisomal biogenesis factor 10, which is typically associated with various neurodevelopmental disorders (Régal et al. 2010). A phenotype characterized by ataxia and neuropathy caused by *PEX10* has previously been reported (Régal et al. 2010; Renaud et al. 2016). Notably, an adult patient with a compound heterozygote variant, including a start‐loss variant c.2T> C:p.(Met1?) was reported, which was subsequently shown to skip the first initiation codon (Régal et al. 2010). To date, only a few patients have been described as having *PEX10‐*neuropathy, and this is the first case being reported in a population of African descent. Additionally, our patients presented Benign Adult Familial Myoclonic Epilepsy type 8 (BAFME8), caused by a pentanucleotide expansion and insertion in the *RAI1* gene, which may have altered the classical presentation of *PEX10*‐neuropathy in these individuals (Yeetong et al. 2024). In fact, the ataxia phenotype in this family may be linked to BAFME8 rather than being a variant of PEX10‐related neuropathy.

## 5 Conclusion

We report rare cases of CMT subtypes in an SSA population for the first time caused by rare variants in *BAG3*, *BSCL2, PEX10*, and *SH3TC2* genes. This study highlights the need for multicentric and collaborative studies on CMT in Africa to uncover rare variants that could have implications for other populations and potentially lead to therapeutic advancements for these degenerative diseases on the continent.

## Author Contributions

**Abdoulaye Yalcouyé**: data curation, formal analysis, investigation, methodology, validation, visualization, writing–original draft, writing–review and editing**. Lassana Cissé**: investigation, formal analysis, writing–review and editing**. Salimata Diarra**: data curation, formal analysis, investigation. **Seybou H. Diallo**: investigation. **Salia Bamba**: data curation, formal analysis. **Patra Yeetong**: investigation, formal analysis. **Boubacar Maiga**: investigation. **Kékouta Dembélé**: investigation. **Dramane Coulibaly**: investigation. **Salimata Diallo**: investigation. **Abdoulaye Taméga**: investigation. **Alassane Baneye Maiga**: investigation. **Hamidou O. Ba**: investigation. **Vorasuk Shotelersuk**: investigation, resources, formal analysis. **Kenneth H. Fischbeck**: conceptualization, resources, methodology. **Cheick O. Guinto**: conceptualization, resources, supervision, methodology. **Guida Landouré**: data curation, formal analysis, investigation, methodology, validation, visualization, funding acquisition, writing–review and editing.

## Ethics Statement

We confirm that we have read the Journal's position on issues involved in ethical publication and affirm that this report is consistent with those guidelines.

## Conflicts of Interest

The authors declare no conflicts of interest.

### Peer Review

The peer review history for this article is available at https://publons.com/publon/10.1002/brb3.70496

## Supporting information




**Figure S1**: Secondary structure prediction of mutated proteins in CMT. **A)** wildtype and **B)** mutant structures of BAG3 showing the changes due to the mutation (black boxes) and the location of the variant (red boxes).

**Table S1**: List of genes included in the next‐generation sequencing panel testing
**Table S2**: Characteristics of the variants in *BAG3*, *BSCL2*, *SH3TC2*, *PEX10* genes

Supporting Information

## Data Availability

The data supporting the current study are available from the corresponding author upon reasonable request.
