# Supplementary figures and images for: Rare Variants Cause Charcot‐Marie‐Tooth Disease in Malian Families
Source: Brain Behav. 2025 May 5;15(5):e70496. doi: 10.1002/brb3.70496 (PMC12050408; doi:10.1002/brb3.70496)

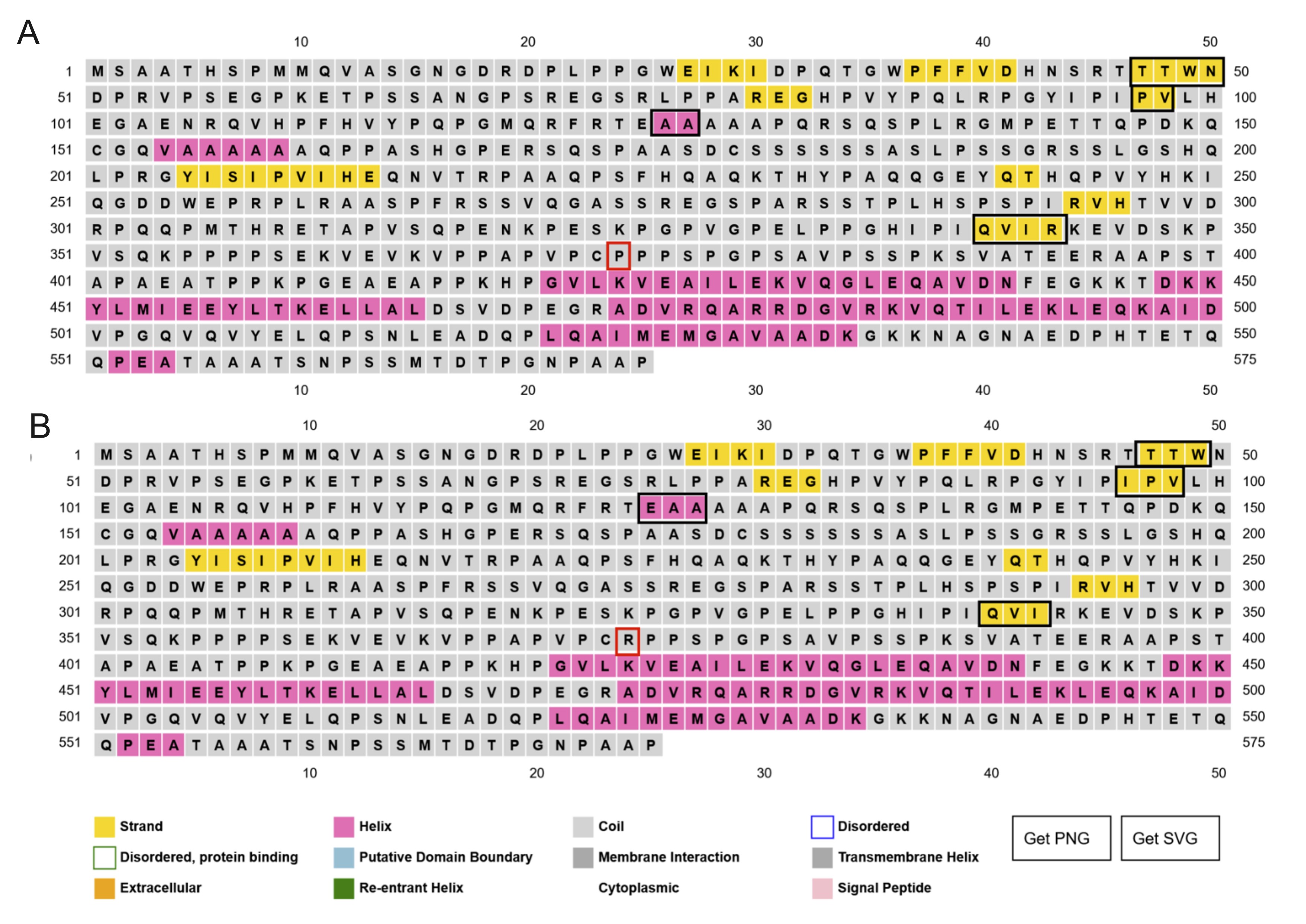

Supplement: Supplementary file 1 — Figure S1: Secondary structure prediction of mutated proteins in CMT. A) wildtype and B) mutant structures of BAG3 showing the changes due to the mutation (black boxes) and the location of the variant (red boxes). [file BRB3-15-e70496-s001.jpg]
