# Supplementary material for: Rare Variants Cause Charcot‐Marie‐Tooth Disease in Malian Families
Source: Brain Behav. 2025 May 5;15(5):e70496. doi: 10.1002/brb3.70496 (PMC12050408; doi:10.1002/brb3.70496)
Supplement: Supplementary file 2 — Table S1: List of genes included in the next‐generation sequencing panel testing Table S2: Characteristics of the variants in BAG3, BSCL2, SH3TC2, PEX10 genes [file BRB3-15-e70496-s002.docx]

**Table S1**: List of genes included in the next-generation sequencing panel testing

| **GENES** | **OMIM ID** |
| --- | --- |
| *AARS* | 601065 |
| *AIFM1* | 300169 |
| *ARHGEF 10* | 608236 |
| *BAG3* | 603883 |
| *BSCL2* | 606158 |
| *C120rf65* | 613541 |
| *COX6A1* | 602072 |
| *CTDP1* | 604927 |
| *DHTKD1* | 614984 |
| *DNM2* | 602378 |
| *EGR2* | 129010 |
| *FBLNS5* | 604580 |
| *FGD4* | 611104 |
| *FIG4* | 809390 |
| *GAN* | 805379 |
| *GARS* | 800287 |
| *GDAP1* | 606598 |
| *GJB1* | 304040 |
| *GNB4* | 610863 |
| *HINT1* | 601314 |
| *HK1* | 142600 |
| *HSPB1* | 602195 |
| *HSPB8* | 608014 |
| *INF2* | 610982 |
| *KARS* | 601421 |
| *LITAF* | 603795 |
| *LMNA* | 150330 |
| *LRSAM1* | 610933 |
| *MED25* | 610197 |
| *MFN2* | 608507 |
| *MPZ* | 159440 |
| *MTMR2* | 603557 |
| *NDRG1* | 805262 |
| *NEFL* | 162280 |
| *PDK3* | 602526 |
| *PLEKHGS* | 611101 |
| *PMP22* | 601097 |
| *PRPS1* | 311850 |
| *PRX* | 605725 |
| *RAB7A* | 602298 |
| *SBF1* | 603560 |
| *SBF2* | 607697 |
| *SEPTO* | 604061 |
| *SH3TC2* | 608206 |
| *SLC12A6* | 604878 |
| *TFG* | 602498 |
| *TRIM2* | 614141 |
| *TRPV4* | 605427 |
| *VCP* | 601023 |
| *YARS* | 603623 |

| **Prediction tools** | ***BAG3*: p.Pro374Arg** | ***BSCL2*: p.Asn88Ser** | ***SH3TC2*: Arg1109Ter** | ***PEX10*: p.Met1?** |
| --- | --- | --- | --- | --- |
| [BLOSUM](https://en.wikipedia.org/wiki/BLOSUM) | Uncertain | Uncertain | - | - |
| [CADD](https://cadd.gs.washington.edu/) | 19.9 | 25.4 | 20.3 | 21.1 |
| [DANN](https://pubmed.ncbi.nlm.nih.gov/25338716/) | Benign | Damaging | Benign | Benign |
| [DEOGEN2](http://deogen2.mutaframe.com/) | tolerated | Uncertain | - | Benign moderate |
| [EIGEN](http://www.columbia.edu/~ii2135/eigen.html) | Benign | Damaging | Moderate | Benign Moderate |
| [EIGEN PC](http://www.columbia.edu/~ii2135/eigen.html) | Benign | Damaging | Moderate | Benign Moderate |
| [FATHMM](http://fathmm.biocompute.org.uk) | tolerated | Damaging | - | Uncertain |
| [FATHMM-MKL](http://fathmm.biocompute.org.uk/fathmmMKL.htm) | Damaging | Damaging | Damaging | Benign Moderate |
| [FATHMM-XF](http://fathmm.biocompute.org.uk/fathmm-xf/) | Neutral | Damaging | Damaging | Benign |
| [LIST-S2](https://precomputed.list-s2.msl.ubc.ca/) | Tolerated | Pathogenic | - | - |
| [LRT](http://www.genetics.wustl.edu/jflab/lrt_query.html) | deleterious | Pathogenic | Deleterious | Uncertain |
| [M-CAP](http://bejerano.stanford.edu/MCAP/) | Benign | Damaging | - | Pathogenic |
| [Mutation assessor](http://mutationassessor.org/r3/) | Medium | Medium | - | - |
| [MutationTaster](http://www.mutationtaster.org/) | Disease causing | Disease causing | - | Uncertain |
| [MutPred](http://mutpred.mutdb.org/) | Benign | Pathogenic | - | Uncertain |
| [MVP](https://github.com/ShenLab/missense) | Uncertain | Pathogenic |  | Benign moderate |
| [PolyphenCat](http://genetics.bwh.harvard.edu/pph2/) | - | - | Probably damaging | - |
| [PrimateAI](https://github.com/Illumina/PrimateAI) | Tolerated | Tolerated | - | - |
| [PROVEAN](http://provean.jcvi.org/index.php) | Damaging | Damaging | - | Benign Moderate |
| [SIFT](http://provean.jcvi.org/index.php) | Damaging | Damaging | - | Damaging |
| [SIFT4G](http://sift.bii.a-star.edu.sg/sift4g/public/Homo_sapiens/) | Damaging | Damaging | - | Pathogenic |
| ACMG classification | Unknown significance (PM2, PP1, PP4) | Pathogenic (PP5, PS3, PM1, PM2, PP1) | Pathogenic (PVS1, PM2, PP1, PP4, PP5) | Pathogenic (PVS1, PM2  PP5) |

**Table S2:** Characteristics of the variants in *BAG3*, *BSCL2*, *SH3TC2*, *PEX10* genes
